# Spatio-Temporal Cluster Mapping System in Smart Beds for Patient Monitoring

**DOI:** 10.3390/s23104614

**Published:** 2023-05-10

**Authors:** Mohamed Maddeh, Fahima Hajjej, Malik Bader Alazzam, Shaha Al Otaibi, Nazek Al Turki, Sarra Ayouni

**Affiliations:** 1 College of Applied Computer Science, King Saud University, Riyadh 11451, Saudi Arabia; 2 Department of Information Systems, College of Computer and Information Sciences, Princess Nourah bint Abdulrahman University, Riyadh 11671, Saudi Arabia; 3 Information Technology College, Ajloun National University, Irbid 21163, Jordan

**Keywords:** behavior logs, data analytics, ICT devices, movement activity, network cluster, sensor nodes, spatio-temporal cluster, time series analysis

## Abstract

Innovative technological solutions are required to improve patients’ quality of life and deliver suitable treatment. Healthcare workers may be able to watch patients from a distance using the Internet of Things (IoT) by using big data algorithms to analyze instrument outputs. Therefore, it is essential to gather information on use and health problems in order to improve the remedies. To ensure seamless incorporation for use in healthcare institutions, senior communities, or private homes, these technological tools must first and foremost be easy to use and implement. We provide a network cluster-based system known as smart patient room usage in order to achieve this. As a result, nursing staff or caretakers can use it efficiently and swiftly. This work focuses on the exterior unit that makes up a network cluster, a cloud storage mechanism for data processing and storage, as well as a wireless or unique radio frequency send module for data transfer. In this article, a spatio-temporal cluster mapping system is presented and described. This system creates time series data using sense data collected from various clusters. The suggested method is the ideal tool to use in a variety of circumstances to improve medical and healthcare services. The suggested model’s ability to anticipate moving behavior with high precision is its most important feature. The time series graphic displays a regular light movement that continued almost the entire night. The last 12 h’ lowest and highest moving duration numbers were roughly 40% and 50%, respectively. When there is little movement, the model assumes a normal posture. Particularly, the moving duration ranges from 7% to 14%, with an average of 7.0%.

## 1. Introduction

The Internet of Things (IoT) has made it possible for users to gather and analyze data from a variety of surroundings, gadgets, and other things used in routine everyday activities. IoT items often feature sensors that generate enormous volumes of diverse data. The methods and tools required to analyze this data are provided by the big data analytics discipline [1]. IoT has been employed for both indoor and outdoor things, including household appliances [2], indoor self-location devices [3], and groundwater sensors [4]. IoT has also been utilized in the healthcare industry to monitor patients. IoT, in particular, makes patient remote monitoring feasible. Wearable IoT sensors have several applications in the healthcare industry and have gained a lot of popularity. According to a recent poll, the IoT may significantly improve nursing care in the future [5,6,7]. A thick pressure-sensitive bedsheet was specifically offered by [8] to monitor patients, and from the data gathered, a study of the patients’ sleeping positions was made. Their technique demonstrated accurate posture recognition with a reliability of 83%. Additionally, a system for sensing was presented by Garca-Magario et al. [9]. To the best of the authors’ knowledge, however, there is no software simulation framework in the literature that enables researchers to test novel sleeping position detection algorithms without having to use the aforementioned expensive equipment.

In this context, the Internet of Medical Things (IoMT) leverages the Internet of Things (IoT) to enable the gathering and transmission of data from medical devices or sensors, which can then be used to improve patient outcomes [10]. In the context of a smart bed system, IoMT can be used to provide real-time monitoring of a patient’s vital signs, such as heart rate, blood pressure, temperature, and oxygen levels. Additionally, IoMT can enable automated bed adjustments to provide the optimal level of comfort and support for the patient. IoMT can also be used to monitor a patient’s movements and detect any potential issues with bed sores or falls. This can help healthcare providers track a patient, take quick action, and improve patient safety by better understanding a patient’s condition and recommending treatments [11,12,13].

According to a report by Zion Market Research [https://www.zionmarketresearch.com/report/smart-home-market (20 January 2022)], the global smart bed market was valued at USD 6.38 billion in 2019 and is expected to reach USD 18.2 billion by 2025, growing at a compound annual growth rate of 19.6% during the forecast period from 2020 to 2025. The current research in the area of smart beds is mainly focused on developing new technologies and sensors to improve the user experience. However, there is limited research on modeling smart beds for emergency use for patients. The need for smart healthcare services is growing with an aging population, yet there is not enough physical staff in hospitals to meet the need [14]. As a result, improving the service quality and efficiency of hospitals is a crucial problem for society. In this paper, we have identified the primary responsibilities of a physical staff for patients in hospitals and provided a smart bed system. Therefore, it is evident that effective information and communications technology (ICT) device utilization in a smart bed system helps to lessen the burden on care employees. It is proven that by using SVM systems, communication effectiveness for healthcare services can also be improved [15,16].

The smart bed system is a technology-enabled bed that can provide advanced patient monitoring and care. Smart beds can detect when a patient has turned or moved, track vital signs, monitor breathing and heart rate, provide temperature control, and even detect falls or other changes in the patient’s condition. Smart beds can also be used to provide personalized comfort, support, and healing. Smart bed systems can be managed and controlled remotely, providing caregivers with greater visibility into a patient’s condition and allowing them to take action quickly in the event of an emergency. Additionally, smart beds can be connected to other medical devices, such as oxygen monitors and infusion pumps, to help streamline patient care [17]. Direct operations, however, are not often the main duties of a smart bed system. The smart bed system mainly identified indirect activities only. Additionally, the smart bed system infrastructure is greatly influenced by the quality of indirect activities. Improvement of indirect operations is thus essential for any smart bed service defined.

Use logs of ICT device-based systems are evaluated in this work. These logs are automatically gathered into the SVM system’s database. An animation tool that visualizes prior operations as well as the placement and movement of patients is suggested in [18] to aid in discussions about the assessment of their performance. Finding information manually that can help with progress, however, takes a lot of time. The user experience may thus be enhanced by using data mining methods to automatically extract important information. The usage logs are used in [19] to apply a data mining approach that has been avidly researched in the fields of outlier detection [20] and anomaly detection [21] for the purpose of identifying suspicious individual behaviors of patients at the bed. However, given the sort of services that require teamwork from numerous people, reviewing and analyzing previous teamwork is crucial to enhancing quality.

In this article, we place a greater emphasis on the collective behavior of patients and provide a technique for identifying operational capabilities for a smart bed system throughout the whole service area using ICT device usage records.

Since each ICT gadget collects usage logs individually, data fusion is necessary when working with different patients. The usage log analysis is done with the idea of situation awareness (SA) [22] in mind.

**Figure 1:**
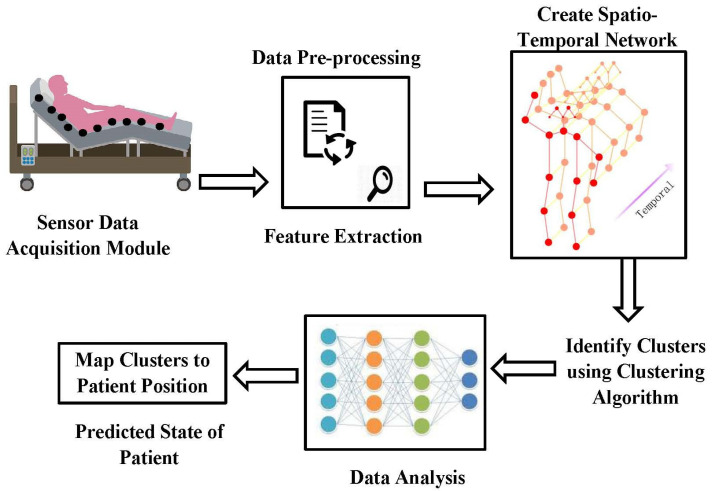
Schematic of the proposed model.

The general concept of the suggested strategy is presented in [23] by the authors, but the specifics of the method and a discussion of the findings are not included. In this study, we demonstrate the suggested method’s step-by-step workflow. Figure 1 shows the schematic of the proposed model. Furthermore, the outcomes of the method’s application to actual data collected at a hospital are carefully examined.

The structure of the paper is as follows. The setting of bed regions and event logs is given in Section 2, which describes the modeling of smart bed parameters from behavior record logs. The suggested scenario for record logs data analytics is provided in Section 3. The results and discussion, including validation of record logs behaviors, are detailed in Section 4, and finally, Section 5 concludes the paper.

## 2. Bed Regions and Event Logs

**Figure 2:**
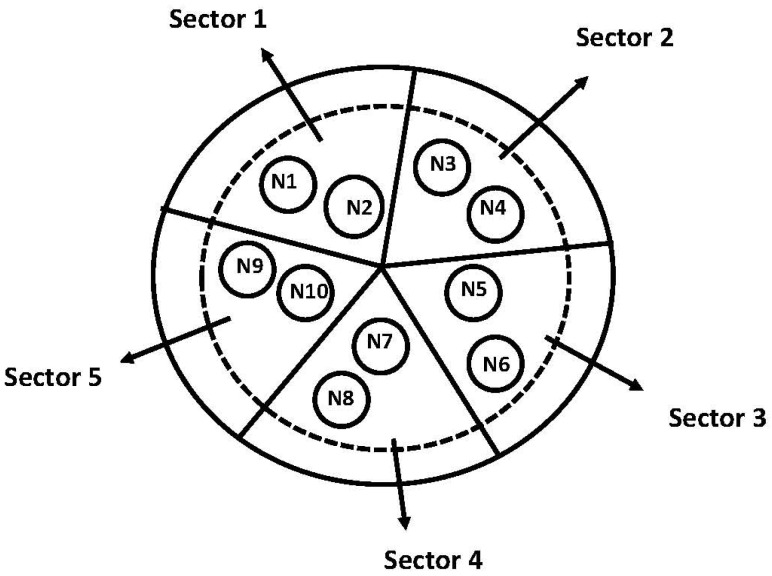
Smart bed region with 10 nodes and 5 sectors.

During field research at a hospital, ICT gadget use records are gathered from a patient’s room. The first goals of the tests were to assess the system’s efficiency and confirm that crucial information is accurate. The smart bed region is split into 10 nodes for further investigation, as shown in Figure 2, which makes a network cluster for further investigation. The smart bed is divided into five sectors, each with two sensor nodes allocated. Each of these terminals behaves like a node, generating some data. Each node is associated with a sensor for heart rate monitoring, a blood pressure monitoring sensor, a pressure sensor for the smart bed, and a vector sensor to record the movement of the patient.

Patient locations on the bed are gathered during the tests and entered into a database. We solely consider the patient’s location data in this study.

Every time there is a change in the position of a patient, the location information is logged. We refer to them as behavior logs. The raw data are sent to the server from each SVM terminal, which receives signals from the Bluetooth markers. Following data processing, the outcome is noted in the format where Type is either the location or “moving”, and the other variables are Time and Duration. The personnel do not remain at any point throughout the moving events. The length of the time period is noted for the “moving” type [16].

**Figure 3:**
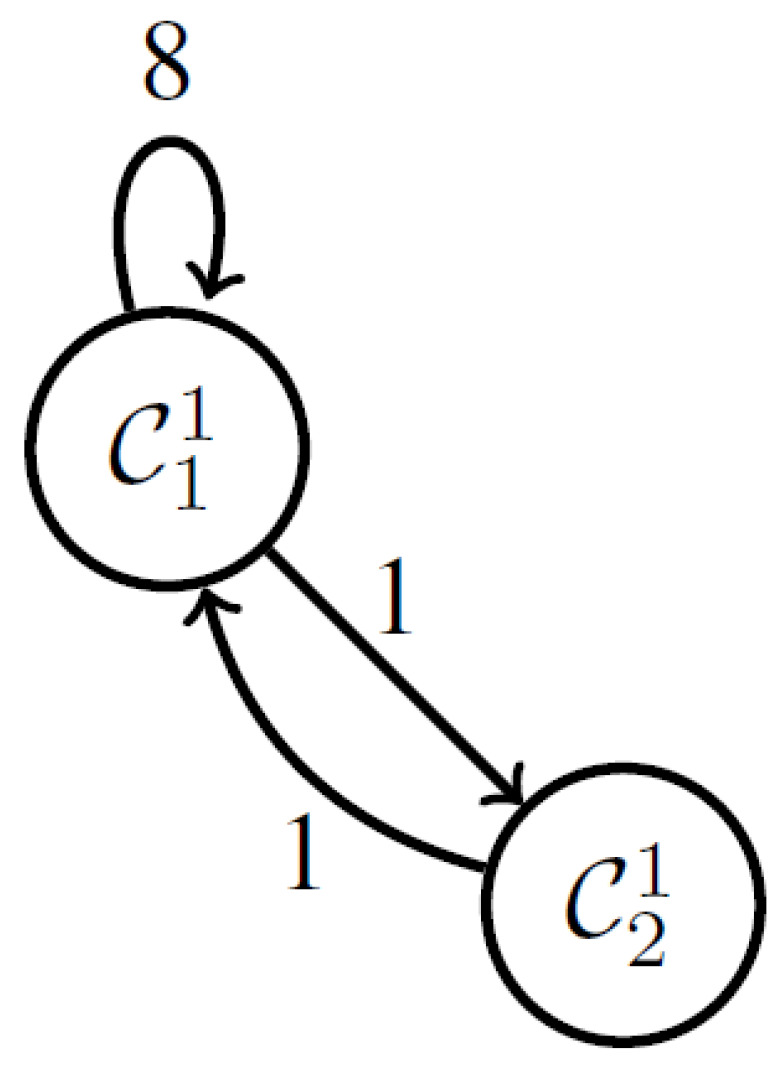
Network cluster with less movement.

**Figure 4:**
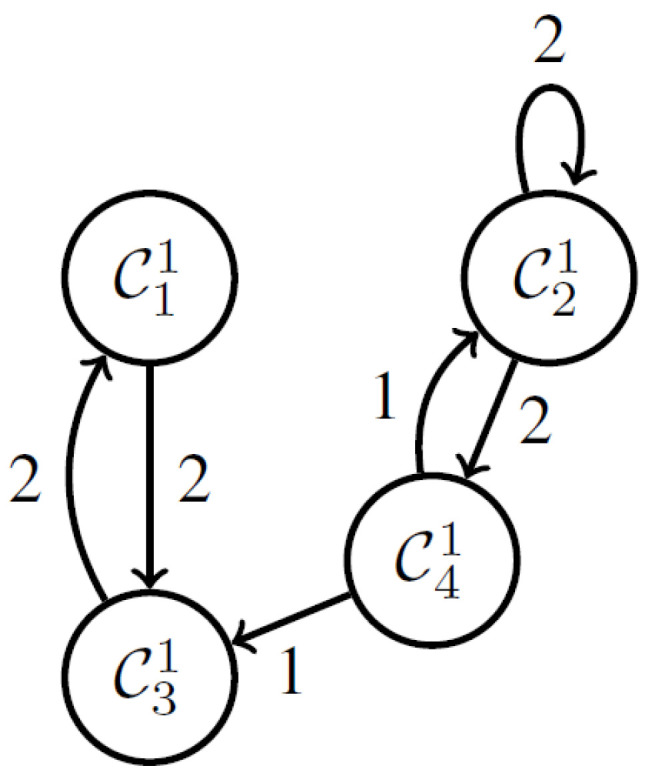
Network cluster with more movement.

For instance, Figure 3 and Figure 4 show the smart bed arrangements based on patient movement for the circumstances using just the behavior logs as less movement and more movement, respectively. The biggest challenge is figuring out how to recognize a scenario such as the one in Figure 4 when movement increases as the network cluster size grows.

### 2.1. Modeling Smart Bed Parameters from Behaviour Record Logs

A patient’s need for help may be determined using the bed parameter readings from the behavior record logs. Our smart bed technology is generally built to send clinical data in real time. Conventional data collection techniques and approaches find it difficult to manage and retain such enormous volumes of data. The suggested design uses a variety of linked smart bed gadgets to obtain clinical data. Wearable sensors are linked to the smart bed in order to collect physiological data in order to make decisions in time-sensitive scenarios. Table 1 describes the various sensors attached to each sensor node.

**Figure 5:**
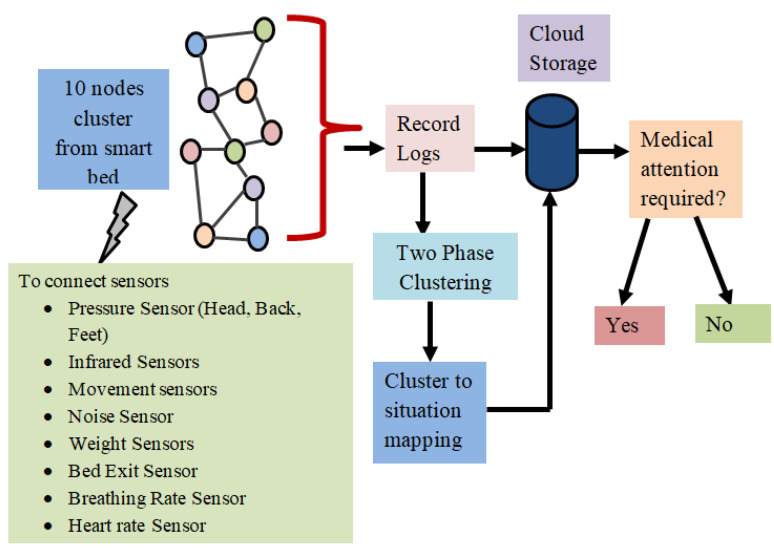
Architecture of the proposed model connected to smart bed system.

Pseudocode 1 presents the stepwise procedure for handling pressure sensor data by network cluster, and Pseudocode 2 presents the stepwise procedure for handling (infrared sensor, motion sensor, weight sensor, bed exit sensor, breathing rate sensor, and heart rate sensor) data by network cluster. Pseudocode 3 presents the cloud storage process as mentioned in Figure 5.

#### 2.1.1. Pseudocode 1: Pseudocode for Handling Pressure Sensor Data by Network Cluster

  # Input: Array of pressure sensors

  # Output: Array of clustered pressure sensors

  # Create an empty array for the clusters

  **Clusters = []**

  # For each pressure sensor in the array:

  **FOR each sensor IN Array:**

  # Create a cluster with the current pressure sensor

  **new_cluster = [sensor]**

  # For each pressure sensor in the array: FOR each other_sensor IN Array:

  # If the pressure sensor is within a certain distance of the current pressure sensor:

  **IF distance (sensor, other_sensor) < threshold:**

  # Add it to the current cluster

  **new_cluster.append (other_sensor)**

  # Add the cluster to the list of clusters

  **clusters.append (new_cluster)**

  # Return the list of clusters

**RETURN clusters**

#### 2.1.2. Pseudocode 2: Pseudocode for Handling (from Infrared Sensor, Motion Sensor, Weight Sensor, Bed Exit Sensor, Breathing Rate Sensor and Heart Rate Sensor) Data by Network Cluster

  # Initialize a variable to hold the sensor data

  **var sensorData;**

  # Loop through all the data from the sensor

  **FOR each data from the sensor**

  # Store the data in the sensorData variable

  **sensorData = data;**

  **IF data is within the range**

  # Send the sensorData to the Network Cluster

  **Send sensorData to Network Cluster**

  **IF the data is not within the range**

  # Generate an alert

**RETURN sensorData**

#### 2.1.3. Pesudocode 3: For Cloud Data Storage

# CLOUD STORAGE process (Datacf, Dataws)

# Install the gcloud component that comes with the Python AppEngine extension:

**gcloud elements: app-engine-python package**

# Put Google Cloud Storage in assemble a bucket

**Upload information from smart bed gadgets to the bucket.**

# Transfer clinical form data to the bucket in step k.

# A program to export local files to Google’s cloud storage.

**Import cloud storage**

**Customer = storage**

**Client (project = ‘proj1’)**

**‘Proj1 bucket’ in client.get bucket;**

**Bucket. Blob (‘proj1.csv’); blob2**

**Blob2.upload from filename (filename = ‘Y: /proj1 data.csv’)**

End of process

Figure 5 provides an illustration of the suggested approach’s concept. There are different nodes considered for smart bed monitoring. It is vital to take into account (i) how the nodes are distributed on the smart bed and (ii) how the node arrangement may vary over time in order to determine the patient’s position.

The suggested technique’s complete process, which is based approximately on the feature-based method of time series clustering [24], is comprised of the following two steps: (1) mapping clusters to scenarios after feature extraction from the behavior logs; and (2) spatial-temporal clustering. The specifics of these actions are discussed in the remaining paragraphs of this section.

**A.**  **Extraction of Features from the Behavior Logs**

In the first stage, the behavior logs are used to extract the patient’s placement on the bed and represent it as a vector. The bed is divided into five sectors with 10 nodes. The place vector’s components are represented by each of the five regions, and the value of each component indicates how many times the patient changed positions. When a person moves from one position to another, the value is divided in half and added to both the start and destination places. The place vector [0, 0.5, 0, 3, 2.5, 0, 0, 0, 0, 0], for instance, represents a scenario where three time positions are changed that are located in area 1, two time positions are changed that are located in area 3, and one time position is changed that is located from area 3 to area 4. We acquire a time series of these location vectors that correspond to the patient’s position at a certain moment in time.

**B.**  **Spatial-Temporal Clustering**

The collection of place vectors is subjected to two-phase clustering. Algorithm 1 presents the steps for the proposed spatio-temporal clustering. The first phase of clustering concentrates on the geographic component of the data, whereas the second phase concentrates on the time component. In the first phase, the location vectors are divided into several sets of vectors based on a similarity metric. The goal of this stage is to categorize the special attention service for patients. The weighted directed graphs depict the transition of the staff service for patients, which is clustered in the second phase. This grouping is used to categorize patient-moving actions. The clustering methodology is based on the Ward method [25], one of the hierarchical clustering techniques.
**Algorithm 1: Algorithm for the proposed spatio temporal clustering****Input**: First phase place vector**Output**: Output clusters in the second phase clustering (in this work it is limited to 5).

**Step 1: *First-phase Place Vector Clustering:*** In this approach, the collection of place vectors is divided into a number of clusters. When the appropriate number of clusters, which is determined by a priori information, is reached during clustering, cluster merging is halted.

**Step 2: *Graph Generation:*** From the collection of clusters acquired in the previous phase, weighted directed graphs are built in this stage. Each graph shows how the patient’s placement has changed throughout time. The specific steps are explained as follows:

**Step 2.1:** The location vector time series is converted into a time series of the first phase clustering’s discovered clusters.

  ● Let $C^{1}(p)$ stand for the cluster that includes a place vector p, and let $p_{t}$ stand for the place vector at time t.
  ● The time series of location vectors $p_{t_{1}}$ and $p_{t_{2}}$ is then translated into a time series of clusters $C^{1}(p_{t_{1}})$ and $C^{1}(p_{t_{2}})$, and finally into a time series of two.
  ● Each interval on the timeline has a length, which is a predetermined parameter.
  ● Each time period of length is further broken down into n subintervals, where n is likewise a predetermined integer.

**Step 2.2:** The representative cluster is then chosen for each sub-interval, where the representative cluster is the one that spends the majority of the sub-interval together.

  ● At the $i^{th}$ outer interval, we obtain the time series of the following n clusters: $C_{i_{1}}^{1},C_{i_{2}}^{1},\ldots ,C_{i_{n}}^{1},$ Be aware that some of the clusters in this series could be repeated.

**Step 2.3:** Finally, the transition of clusters in the time series is used to create the edge-weighted directed graph G=(V,E), where $V=\left\{C_{i_{1}}^{1},C_{i_{2}}^{1},\ldots ,C_{i_{n}}^{1}\right\}$ is the collection of nodes, and E:V×V→N is a function that represents the quantity of edges between two nodes such that $E(C_{i}^{1},C_{j}^{1}):=\left|\left\{k\in \{1,\ldots ,n\}|C_{i}^{1}=C_{i_{k}}^{1},C_{j}^{1}=C_{i_{k+1}}^{1}\right\}\right|$.

  ● According to the definition, if $k=n,C_{i_{n+1}}^{1}$ denotes $C_{i+1}^{1}$ which is the first cluster in the next interval.
  ● A change from $C_{i}^{1}$ to $C_{j}^{1}$ in the time series is represented by an edge from node $C_{i}^{1}$ to $C_{j}^{1}$. The number of these transitions is counted by the function E.
  ● The total of the edge weights is the same amount n for whatever duration of time. The graphs are normalized in this way. On the other hand, the number of nodes could vary. Note that most edges form self-loops in situations with little movement.

**Step 3: *Directed graph second-phase clustering:*** There are many graph similarity metrics that may be used for clustering [26,27,28]. Here, we only choose the adjacency matrix’s similarity [29,30,31]. The adjacency matrix $A=[a_{ij}]$, where $a_{ij}$ represents the number of edges connecting node i to node j, serves as a representation for each directed graph. For the collection of all adjacency matrices, the Ward technique is used. The number of clusters is chosen similarly to the first phase, such that derived clusters include all different kinds of observed instances.

**Step 4:** The clusters are obtained in the second-phase clustering as $C_{i}^{2},i=0,1,\ldots .$.

**C.**  **Cluster to Situation Mapping**

The last stage involves mapping the generated clusters from the second phase into known operational scenarios. By examining the characteristics of derived clusters and operating scenarios, the mapping is carried out.

## 3. Record Logs Data Analytics

**Figure 6:**
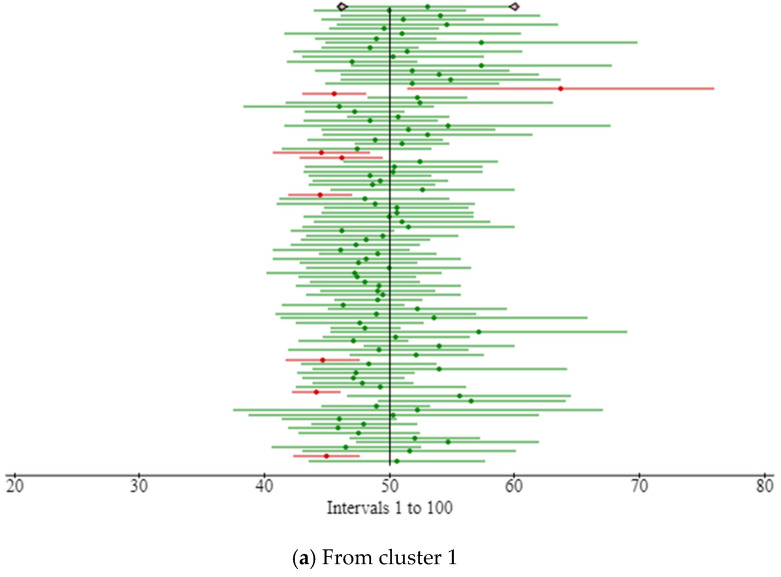

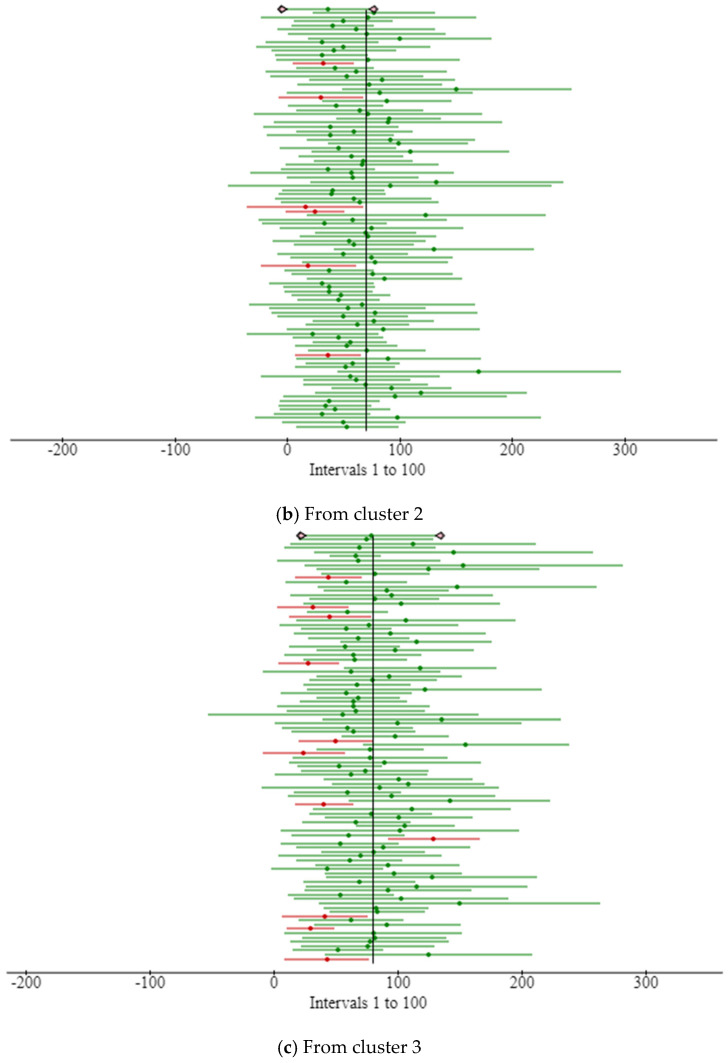

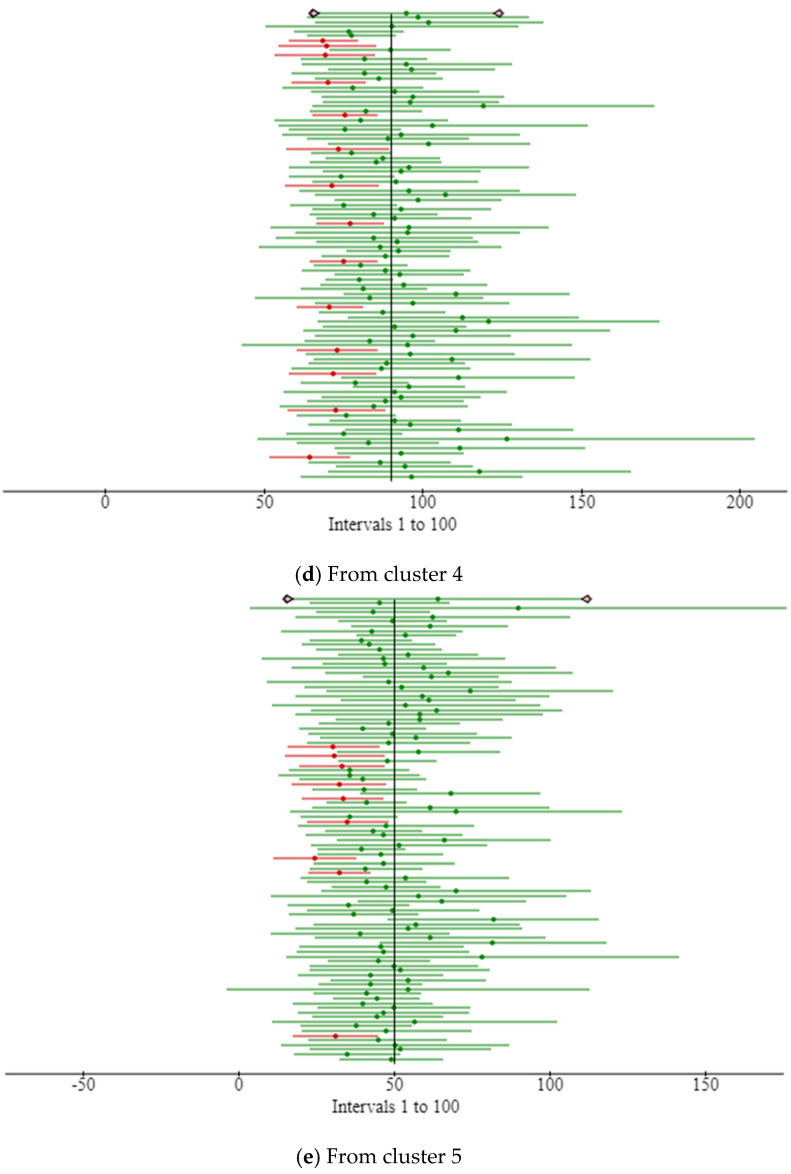
Record logs progression from the sensor nodes (average of all nodes): (**a**) from cluster 1; (**b**) from cluster 2; (**c**) from cluster 3; (**d**) from cluster 4; (**e**) from cluster 5.

The prescribed network cluster platform is created to investigate big data analytics within the IoT environment, especially in the context of smart beds. It is possible to simulate the movement on the bed and record the sensor states throughout the course of the simulation. A log file is where this data is kept. This attribute is internally configurable based on the class file. The created file includes data from all the sensor nodes for each minute that are associated with the patient’s position on the bed and their orientation (such as frontal or lateral). Figure 6 presents the record logs progression from the sensor nodes (average of all nodes). In this figure, the x-axis is node output measured between two consecutive nodes. The node input and node output are the signals from sensor nodes that quantify the patient’s movement. We have considered here the average of all sensor nodes. If the movement is logged high on a sensor node, the magnitude is logged high; otherwise, it is logged low. Most investigations have used at least a pair of sensor nodes. A dataset in a public research repository contains examples of these created logs [32]. Each simulation has a significant number of nondeterministic choices, which leads to the majority of the simulations being distinct from one another. This volume of data may be helpful for learning about big data analytics approaches and progressing in the creation of algorithms for the analysis of large amounts of data from smart bed sensors.

### Cluster Strategy

The following conditions are used to apply the strategy to the usage logs received from the bed nodes in order to assess the efficiency of the suggested approach:

● We utilize the usage logs for three days. There are six behavior logs in total each day.
● There are now six nodes employed across all of the use logs.
● Eight clusters will be used in the initial round of clustering.
● The graph building step’s time interval length and number of sub-intervals are both set to 200 s and 10, respectively.
● Five clusters will be used in the second step of clustering.

**A.**  **Initial stage Clustering**

There are 1057 place vectors, and after grouping, we get 8 clusters.

**Figure 7:**
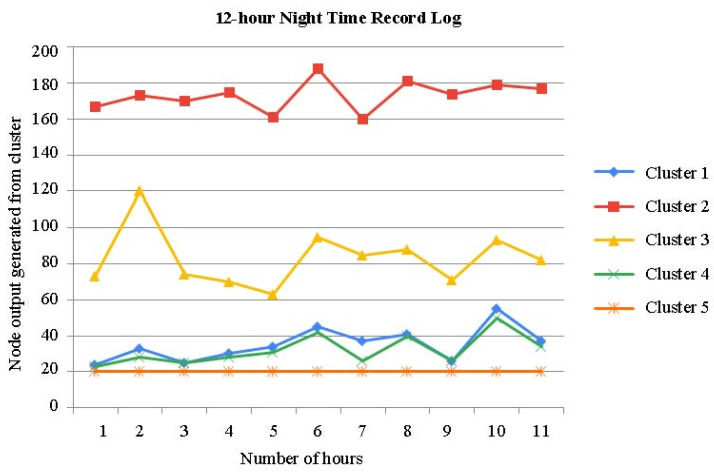
Time series analysis: 12 h night time record log of patient bed of five clusters.

The time series of the aforementioned clusters during the night time is shown in Figure 7. It has been noted that the clusters do not clearly delineate the time series, particularly before and after movement.

**B.**  **Phase two Clustering**

One hundred and six graphs in all are created. The adjacency matrices of these graphs are then subjected to the Ward approach. By carefully examining the produced clusters, we may draw the following conclusions:

● $C_{1}^{2}$: The graphs are made up of self-loops from $C_{1}^{1},C_{2}^{1},C_{6}^{1},$ and $C_{7}^{1}$.
● $C_{2}^{2}$: Graphs are mostly made of self-loops from $C_{4}^{1}$
● Graphs in $C_{3}^{2}$ are mostly made up of self-loops from $C_{3}^{1}$.
● $C_{4}^{2}$: Self-loops from $C_{5}^{1}$ make up the majority of the graphs.
● Graphs in $C_{5}^{2}$ are mostly made up of self-loops from $C_{8}^{1}$.

**Figure 8:**
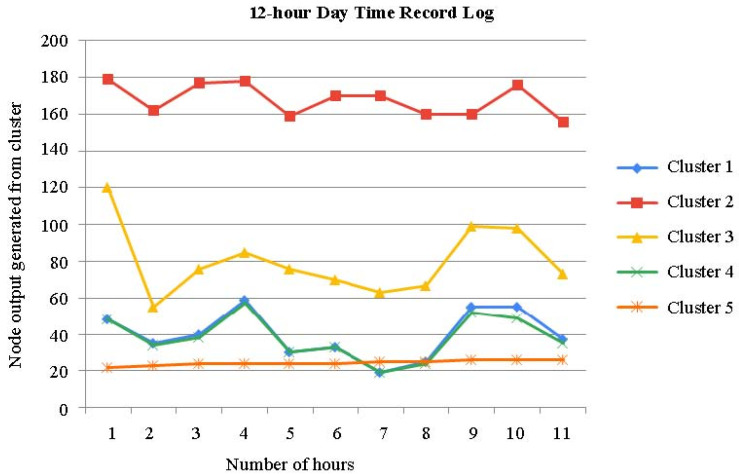
Time series analysis: 12 h day time record log of patient bed of five clusters.

The time series of the aforementioned clusters during the day time is shown in Figure 8. When compared to Figure 7, it can be seen that the clusters more clearly demarcate the chronology.

**C.**  **Cluster to Situation Mapping**

Based on an examination of the time series of clusters and their center vectors, derived clusters are mapped into recognized operational circumstances. The findings of the analysis are summed up as follows.

● $C_{1}^{2}$: This cluster often manifests around the start of the movement. Because this cluster incorporates four distinct clusters from the first phase of clustering, it may regularly relocate to new locations.
● $C_{2}^{2}$: During morning and night, this cluster often manifests. Areas 7 and 8 are divided equally; however, region 8 has a larger population than area 7.
● $C_{3}^{2}$: This cluster depicts a scenario where Area 8 has maximum movement.
● $C_{4}^{2}$: The first part of the morning and night is when this cluster often manifests. Areas 7 and 8 are divided equally.
● $C_{5}^{2}$: This cluster often manifests at the conclusion of the night.

If there is a correlation between clusters and operational conditions that we are already aware of, we may automatically determine the operational state from a behavior record.

## 4. Results and Discussion

**Figure 9:**
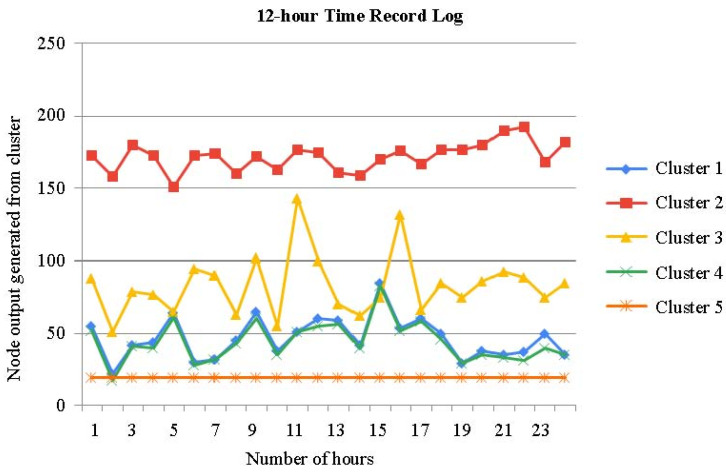
Time series analysis: 24 h time record log of patient bed with a log of five clusters.

There are times when clusters transit often in the time series of the clusters acquired in the first phase, and some clusters are dispersed across the other figures (Figure 7 and Figure 8). Figure 7, Figure 8 and Figure 9 show a time series analysis of data from the smart bed, which has been recorded as the 12 h–24 h activity log from the smart bed. In Figure 7, Figure 8 and Figure 9, the x-axis is the time in hours, and the y-axis is the node output generated for each cluster. In the time series of the clusters in the second phase, one cluster occupies each period of the time axis, and dispersed clusters essentially vanish. As a result, the second-phase clustering could help with the first-phase clustering’s issue, which is that scenarios involving mobility are not only determined by the patient’s placement.

However, there are still some issues with the suggested approach. The fact that the location vectors are deterministically grouped and that the outcomes of the first phase of clustering heavily influence the second-phase clustering may be due to this: For the purpose of transferring information into the graph creation step, the spatial properties are oversimplified. Instead of using hierarchical clustering, it is beneficial to employ a fuzzy clustering technique.

Additionally, during the graph formation phase, types of edges connecting many nodes have a higher volume than kinds of self-loops from a single node. As a result, changes in patient position are not accurately represented in the second-phase clustering. Since such flow information is one of the key indicators used to discern between working staff conditions, this issue needs to be addressed in our ongoing research.

The suggested approach is also intended to be utilized for anomaly detection. If the present directed graph differs from previously known ones, an abnormality in the patient is likely to have occurred, and immediate medical attention is required.

### Validation of Record Logs Behaviors

We offer several instances of the simulated evolutions of the various movement activities in order to officially demonstrate if they behaved as predicted. Figure 7, Figure 8 and Figure 9 illustrate a step-by-step progression of the patient’s movement along the time axis. Even though these stances varied from simulation to simulation, i.e., when the movement is recorded above 30%, it is usually assumed that attention is needed by the patient. The key characteristic of the proposed model is its high accuracy in predicting movement activity. A light movement that lasted virtually the whole night with frequent movements can be seen in the time series chart. The lowest and greatest movement time values for the last 12 h were around 40% and 50%, respectively. The standard deviation is 5.5%, while the average is 54% long term. When movement is limited, the majority of the time it assumes a regular position. The movement time, in particular, varies from 7% to 14%, with an average of 7.0%.

With the use of node clustering and the big data produced by the sensor nodes, the current strategy has provided a framework for enabling researchers to test algorithms in a simulated smart bed. We employed two distinct estimators based on two-phase clustering. We conducted the studies and discovered that the attribute that may have the most detrimental impact on the estimator’s capacity to identify the patient’s movement when it changes randomly. Due to the simulations’ increased realism and wide range of scenarios, we chose to preserve these random motions. These changes also provide the potential for enhancing the algorithms that smart beds use to accurately recognize the positions of the sensor nodes. Thus, in the future, other researchers might test more sophisticated algorithms in the context of big data analytics using the created logs.

The present method makes it simple to test smart beds with various sensor nodes to accurately identify patient positions, allowing for the construction of inexpensive smart beds in the real world. To gain better trade-offs between accuracy and cost, the presented smart bed system may need to be expanded to take into account sensor layouts other than grids.

## 5. Conclusions and Future Work

Despite the fact that there are several scenario detection methods for spatio-temporal data, our primary emphasis is on the movement of individuals. The scenario is specified for a group of clusters, which is the major contribution of the suggested technique. Even though the suggested technique mainly succeeds in capturing the spatio-temporal characteristics of the operational circumstances, there is still potential for improvement. We are thinking of using multi-agent simulation to generate behavior logs since the data is insufficient to validate the suggested strategy.

Future extensions of this work are anticipated in a number of different ways. From the perspective of a big data analytics study, this may be sufficient since the logs capture every movement. However, some end users may want to see an animation of the changes made by the bed sensors. To boost performance, this will be offered as an optional feature. An enhanced future study will provide some user-centered ideas for enhancing the design of large data analyses. Additionally, research will be carried out to identify which grid sensors are really helpful for movement detection in order to achieve a much smaller collection of sensors for this detection. This will lower the price of putting the existing strategy with actual sensors in a smart bed into practice.

## Figures and Tables

**Table 1:** Description of sensors attached to each sensor nodes.

S. No.	Connection to Sensor Node	Type of Sensor	Description about Sensor
1	Node 1	Pressure sensors (head)	Pressure sensors are used to monitor the patient’s pressure points, such as their head.
2	Node 2	Infrared sensors	Infrared sensors are used to detect changes in the patient’s body temperature.
3	Node 3	Movement sensors	Movement sensors measure the amount of movement in the bed, to alert the user if they are tossing and turning too much.
4	Node 4	Noise sensor	Detects loud noises in the bedroom and can be used to alert caregivers or trigger alarms if necessary.
5	Node 5	Weight sensors	Monitors the weight of the bed, allowing for weight distribution and improved comfort.
6	Node 6	Bed exit sensor	This sensor detects when a patient is getting out of bed and triggers an alarm to alert nurses and caregivers
7	Node 7	Pressure sensors (back)	Pressure sensors are used to monitor the patient’s pressure points, such as their back.
8	Node 8	Pressure sensors (feet)	Pressure sensors are used to monitor the patient’s pressure points, such as their feet.
9	Node 9	Breathing rate sensors	These sensors measure the patient’s breathing rate and can alert staff if the patient’s breathing rate changes significantly.
10	Node 10	Heart rate sensors	Heart rate sensors monitor the patient’s heart rate and can alert staff to changes in heart rate.

## Data Availability

Not applicable.
